# Implementation of Person-Centered Care: A Feasibility Study Using the WE-CARE Roadmap

**DOI:** 10.3390/ijerph18052205

**Published:** 2021-02-24

**Authors:** Roman A. Lewandowski, Jędrzej B. Lewandowski, Inger Ekman, Karl Swedberg, Jan Törnell, Heather L. Rogers

**Affiliations:** 1 Management Faculty, University of Social Sciences, 90-113 Lodz, Poland; rlewandowski@san.edu.pl; 2 Voivodeship Rehabilitation Hospital for Children in Ameryka, 11-015 Olsztynek, Poland; 3 Faculty of Medicine, Medical University of Warsaw, 02-091 Warsaw, Poland; jedrzejblew@gmail.com; 4 Centre for Person-Centred Care, Sahlgrenska Academy, University of Gothenburg, 40530 Gothenburg, Sweden; inger.ekman@fhs.gu.se (I.E.); karl.swedberg@gu.se (K.S.); jan.tornell@innoext.se (J.T.); 5 Institute of Health and Care Sciences, Sahlgrenska Academy, University of Gothenburg, 40530 Gothenburg, Sweden; 6 Department of Molecular and Clinical Medicine, Sahlgrenska Academy, 41685 Gothenburg, Sweden; 7 Institute of Neuroscience and Physiology, University of Gothenburg, 40530 Gothenburg, Sweden; 8 Biocruces Bizkaia Health Research Institute, 48903 Barakaldo, Spain; 9 IKERBASQUE Basque Foundation for Science, 48009 Bilbao, Spain

**Keywords:** person-centered care, patient-centered care, implementation feasibility, multi-disciplinary care team, scoliosis

## Abstract

Background: Person-Centered Care (PCC) is a promising approach towards improved quality of care and cost containment within health systems. It has been evaluated in Sweden and England. This feasibility study examines initial PCC implementation in a rehabilitation hospital for children in Poland. Methods: The WE-CARE Roadmap of enablers was used to guide implementation of PCC for patients with moderate scoliosis. A multi-disciplinary team of professionals were trained in the PCC approach and the hospital Information Technology (IT) system was modified to enhance PCC data capture. Semi-structured interviews were conducted with the nine health care professionals involved in the pilot study and three patients/parents receiving care. Transcribed data were analyzed via content analysis. Results: 51 patients and their families were treated via a PCC approach. High proportions of new PCC data fields were completed by the professionals. The professionals were able to implement the three core PCC routines and perceived benefits using the PCC approach. Patients and their families also perceived improved quality care. The WE-CARE framework enablers facilitated PCC implementation in this setting. Conclusions: This feasibility pilot study indicates that the Gothenburg PCC approach can be successfully transferred to a rehabilitation hospital in Poland with favorable perceptions of implementation by both professionals and patients/their families.

## 1. Introduction

Rising costs of healthcare, especially long term care, is an urgent challenge for western countries’ economies. Cost containment, while maintaining quality of care, especially in elective and long term medical services, is an important need. The WE-CARE project (Grant Agreement 602131) funded by the European Union’s (EU) Seventh Framework Programme for Research (FP7) is an important step to tackle these problems. The project developed the WE-CARE Roadmap framework [1] which identified the implementation of Person-Centered Care (PCC) as a vehicle to improve quality and contain the increase of costs in healthcare [2,3], thus improving accessibility and affordability of future health care for all EU citizens.

The WE-CARE Roadmap was the basis for another EU-funded project, COST CARES, COST Action 15222, whose aim is to design a number of Exploratory Health Laboratories (EHLs) to provide scientific evidence on PCC transferability and WE-CARE Roadmap effectiveness across various settings in different EU countries. The original five critical enablers/factors supporting the implementation of PCC to be addressed in the EHLs were: Information Technology, Infrastructure, Incentive Systems, Contracting Strategies, and Quality Measures [1,4]. Within the COST CARES project, and as a result of PCC implementation experience in England [4,5,6], Working Group 3 suggested that Cultural Change also be considered as a sixth potential enabler [7].

PCC differs from evidence-based medicine (EBM) and value-based healthcare (VBH). EBM purports to integrate the best available research evidence with clinical expertise and patient values [8]. In practice, the focus of EBM has been on locating, appraising, and synthesizing scientific evidence, and less on patient involvement [9,10]. This is unlike PCC, in which the patient is a partner in care and an expert who contributes his/her own experience to the evidence [10] Applied, this means that patients are partners and together with professionals plan and perform their care, which have shown to increase self-care, self-efficacy and shorten hospital stay [2,11,12,13]. Another approach to cost-containment is VBH, which is an economic method for measuring, comparing and reporting outcomes in relation to costs [14]. Again, the role of the patient and family member in care and care decisions is not explicitly defined, as it is in PCC [10].

PCC is a promising approach towards improved quality of care and cost containment. It has been evaluated in several health care settings in Sweden and was found to reduce the number of hospitalizations, improve quality of life, and increase self-efficacy (e.g., [6,11,12,13,15]). Given this wealth of evidence, a majority of Swedish regions have decided to implement PCC [16] and there is a growing interest in this PCC model from other countries through the network achieved in the COST CARES project. In this regard, a European standard on patient involvement in health care “Minimum requirements for person-centered care” was approved in April 2020 [17]. The challenge is now HOW to implement PCC in different countries and make the implementation of PCC sustainable for healthcare systems.

This paper aims to explore the value of PCC in a rehabilitation hospital for children that was implemented using the revised WE-CARE Roadmap. This pilot implementation feasibility study addressed two questions:

1. To what extent can the three PCC routines previously tested in Sweden and England be implemented in a rehabilitation hospital for children in Poland?
2. To what extent are the enablers described in the WE-CARE roadmap helpful in this PCC implementation process?

## 2. Materials and Methods

### 2.1. Design, Setting, and Participants

Voivodeship Rehabilitation Hospital for Children in Ameryka, Poland, is a COST CARES pilot EHL. The hospital is located in the Warmia and Masuria region in the north-east part of Poland. It is the biggest rehabilitation center for children in the country. Each year approximately 3000 children with different conditions, including cerebral palsy, craniocerebral trauma and scoliosis, are rehabilitated there.

This pilot feasibility study focused specifically on introducing PCC into rehabilitation care provided to children with moderate scoliosis. This patient population was selected for the pilot phase of PCC implementation for many reasons.

1. The recommendations for scoliosis treatment are, to great extent, standardized, and thus the treatment process and adherence could be consistently monitored. The International Scientific Society on Scoliosis Orthopaedic and Rehabilitation Treatment (SOSORT) recommendations indicate that bracing (primarily the Cheneau corset) must be worn for as much of any 24 h period as possible [18].
2. There is a lack of understanding among the general population about scoliosis and its treatment. Upon diagnosis, young people and their parents need support and psycho-education. In addition, it is often very difficult for young patients to follow the treatment recommendations, especially in the group at high risk of progression that requires Full Time Rigid Bracing, i.e., wearing a corset for a minimum 20 h daily and performing special exercises [18]. However, even for patients with a lower rate of scoliosis progression and less than full-time bracing recommendations, treatment adherence is challenging [19]. Healthcare professionals at the rehabilitation hospital have validated this finding in their own patients. Lastly, scoliosis is often diagnosed at a young age, which means that treatment success depends on the child or adolescent adhering to the treatment plan [20].
3. The number of new patients diagnosed with scoliosis at the hospital was considered to be large enough, but also feasible enough, to effectively start a PCC intervention.

In summary, the number of hours of wearing a corset per day and completion of specific daily exercises in individuals with moderate scoliosis are associated with successful long-term outcomes [20,21]. We hypothesized that implementation of the PCC approach would assist patients and physicians to reach an agreed upon reasonable treatment plan, which would lead to enhanced adherence and better medical outcomes in the long term. This pilot study examines the initial feasibility of PCC approach implementation and its acceptability in a Polish rehabilitation care setting.

Rehabilitation professionals at the hospital heavily involved in scoliosis patient care included four physicians, two physiotherapists, one nurse and one psychologist. All eight of these multi-disciplinary professionals were involved in this pilot study. PCC implementation began in January 2019 and took approximately 10 months to establish. All new patients diagnosed with moderate scoliosis and their family members began receiving PCC in November 2019. To evaluate feasibility and acceptability, interviews were conducted with the involved professionals and three patients/parents who received PCC care in February 2020.

### 2.2. Interventions

#### 2.2.1. PCC Intervention Approach

The Gothenburg model of PCC consists of three pillars, or routines [6,10,22]. Routine 1 involves initiating a partnership by inviting the patient to narrate events from their daily life in relation to their condition, including the sick person’s (often with relatives) description of their illness, their symptoms and their impact. This personal account supplements the medical history that reflects the biomedical markers and evidenced-based guidelines for treating the disease. The strengths that the individual brings to the table are emphasized. Routine 2 encompasses the process of shared decision-making, in which the individual and the multidisciplinary professionals in the partnership create a mutually agreeable care plan that is based on the unique narrative of the patient and the generic knowledge of the professional regarding the course of disease, as well as the anticipated prognosis for the individual. Routine 3 in the PCC process involves safeguarding the partnership by documenting the individual’s narrative and the jointly agreed care plan, and regularly reviewing and updating the documentation.

#### 2.2.2. We-CARE Roadmap

**Figure 1:**
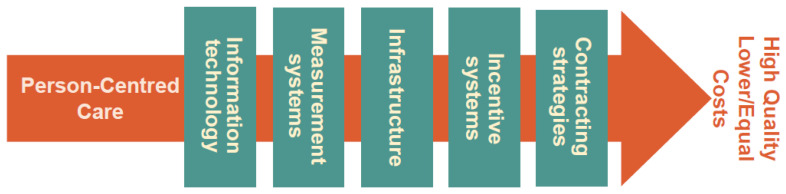
The dimensions and critical enablers of the WE-CARE Roadmap. Source: modified from [7,23].

Figure 1 provides a framework for how PCC could be sustainably implemented and consists of five enablers that interact among themselves:

1. Information Technology (IT) might include IT solutions for communication or wearable devices for monitoring.
2. Infrastructure relates to the necessary resources and structures empowering the delivery of PCC (e.g., hospital organization, home care and their interaction).
3. Incentive systems include all conceivable means which could motivate both patients and professionals to engage in behaviors congruent with PCC.
4. Contracting strategies includes financial solutions and mutual agreements regulating commitments and deliverables.
5. Measurement systems to capture all necessary aspects of the implementation process and its outcome.

Each enabler was evaluated by the implementation team of hospital management and rehabilitation professionals to determine how these enablers might facilitate implementation of PCC with the patient population of interest. Setting-specific strategies targeting the most important WE-CARE enablers discussed in these meetings are described below.

##### Infrastructure

In order to facilitate a team-oriented PCC approach to treatment, some adaptations to physical locations were needed. Remote video-conferencing equipment was installed to enhance communication among the team. Video-conferencing equipment was also used for PCC training. The Gothenburg Centre for Person-Centered Care train-the-trainer online program was used [24], in addition to presentations and distribution of scientific articles. To reinforce patient engagement in PCC, an educational “Scoliosis Activity Diary” was printed. (See Supplementary Materials for Polish-language Diary.) This consisted of cartoons for patients explaining basic information about scoliosis and recommended treatment. Professionals could add personalized tips concerning the treatment and patients could note whether they followed the mutually agreed treatment plan in specific tracking sheets.

##### Information Technologies

Approximately 30 new fields were added to the hospital’s IT system, which includes the electronic medical records (EMR) and cost data. These fields were intended to facilitate implementation of PCC by professionals and to ensure appropriate data capture for monitoring of PCC implementation. The EMR is structured into individual tabs for each type of health care professional, and each tab had a few additional fields. To distinguish “PCC patients”, professionals had to tick a box which opened an additional set of PCC-specific fields. The most important fields were open-ended text fields used by each member of the rehabilitation team to document goals as described by patients and their families, as well as preferences, limitations and capabilities derived from the patient narrative. A field to document the care plan was already part of the EMR prior to PCC implementation.

##### Incentive Systems

Although financial incentives for PCC implementation were considered, this enabler was not implemented in the pilot phase. To be effective, tangible incentives must be based on accurate, objective, and verifiable measures, otherwise, real performance may not be rewarded and unintended consequences could occur [25]. In later stages of implementation, once PCC quality measures have been better defined, this possibility will be re-visited. Scientific articles on the evidence of PCC effectiveness were disseminated to improve motivation [26]. Approximately 3 h of presentations on PCC effectiveness were provided. These dissemination strategies were designed to inspire intrinsic motivation to use PCC in order to improve patients’ medical outcomes, experiences, and satisfaction.

For patients and their families, the “Scoliosis Activity Diary” was implemented to provide more immediate feedback as an incentive for continued corset use. The improvement of the spine core shape in the treatment of scoliosis is very slow. Sometimes the treatment goal is only to prevent further deterioration. Lack of observable improvement can discourage children from adhering to the treatment regimen [19]. The activity diary was designed to allow children self-control and ignite motivation for continuous engagement in the mutually agreed care plan. The booklet was also used to provide individually tailored recommendation for rehabilitation exercises, document challenges and work through potential solutions with the rehabilitation team (See Supplementary Materials for Polish-language copy.).

##### Measurement Systems

Measurement systems were considered from the perspectives of the PCC process implementation and outcomes for short and longer term monitoring. The extent to which professionals followed the PCC approach was measured by the percentage of new key PCC fields completed by each of the team member. The entries (information) from each team member are proxy indicators of the process of shared decision-making and thus the creation of a mutually agreeable care plan. During the implementation process, PCC entries were reviewed and feedback was given to the rehabilitation professionals to enhance treatment fidelity and engagement in PCC routines. The extent to which patients engaged in the mutually agreed care plan was measured using patients’ self-assessments as recorded in the “Scoliosis Activity Diary”. Families and professionals were encouraged not to “control” patient entries into the diary in order to avoid creating potential incentives for over-reporting (e.g., [11]) and diminishing the intrinsic motivation of the child/adolescent to adhere to the agreed upon care plan.

### 2.3. Methods

In February 2020, R.A.L. conducted semi-structured interviews with the nine health care professionals (HCP1–9) involved in the pilot study. Three semi-structured interviews with patients and their parents (P1–3) receiving care in February 2020 were also carried out. Given that this study is considered quality control of an existing PCC standard of care [17], ethical committee approval was not required by the hospital.

The interview guide for rehabilitation professionals asked exploratory questions such as: How did the implementation of PCC change your work routines? What was your role (“the professionals’ role”) in the treatment process? What similarities and differences between PCC and other health-care approaches such as evidence-based medicine did you observe? How did changes in IT, infrastructure, quality measures, and incentives influence implementation of PCC? How did the patients react to the PCC approach? The interview guide for patients and their parents focused on their perceptions of the PCC approach. They were asked to talk about their feelings regarding their inclusion in decision-making concerning the treatment plan, their role and position in the rehabilitation team, and the extent to which they felt they were partners with the professionals. The interviews were conducted in Polish and lasted for an average of 40 min each (range 25–55 min). After the interviews, the most important content was translated into English by J.B.L. and R.A.L. Then, translated materials were sent to the rest of the research team and analyzed using a content analysis approach [27].

## 3. Results and Discussion

### 3.1. Quantitative Data on Feasibility of PCC Implementation

Between November 2019 and September 2020, all 51 new patients with moderate scoliosis seen by this multi-disciplinary team were treated using the PCC approach at Voivodeship Rehabilitation Hospital for Children. These patients were identified according to the marked tick box within the EMR. Table 1 summarizes quantitative findings from this pilot phase of implementation of PCC.

Table 1 shows that very high proportions of patients with moderate scoliosis who were identified as “PCC patients” in the EMR received PCC care. Specifically, each type of health care professional contributed a narrative summary to the open-ended field in the EMR in the overwhelming majority of their patients, between 92% and 100%. This indicates the healthcare professionals’ success at the first PCC of initiating a partnership with the child/adolescent and family regarding goals, preferences, limitations and capabilities. This information was documented into the IT system as part of safeguarding the partnership, part of the third routine. A high proportion of PCC patients, 86%, had a documented treatment plan. The PCC patients without a documented treatment plan were patients “in progress”. Physicians are legally responsible for working out the final treatment plan, in agreement with the patient and other professionals. Thus, after all other specialists put their documentation into the IT system, physicians organize the shared decision-making process and input the treatment plan. The patients without a treatment plan had not yet reached this stage. All PCC patients were given the Activity Diary, which was used by patients and professionals for documentation and feedback concerning the treatment plan. However, as mentioned in the methods section, the Activity Diary served as a self-report tool for patients to see their own progress and the diaries were not analyzed as part of this pilot feasibility study.

### 3.2. Qualitative Data on Implementation of PCC Routines

The semi-structured interviews were analyzed to determine if and how each of the three core routines in PCC had been implemented and the perceptions of changes compared to usual care from both professionals and patients.

#### 3.2.1. From ‘Talking to’, to ‘Talking with’. Creating a Partnership (PCC Routine 1)

A physician described the change of the approach after working with the PCC as follows:

“We have always had the feeling that the patient is standing somehow nearby. That means, the patient stands in the center and we all bend over him as if we impose our will on him. And now (…) after such a change of approach, it seems that the patient joined our interdisciplinary team and the relationship is closer.”

*(HCP1)*

Before the implementation of PCC, professionals talked to patients but did not allow them to genuinely express their perspective. Patients were allowed to talk, but only after some routine questions giving the professional the information he/she believed was needed. The change of mind set involved in a PCC approach towards active listening was described by one professional as:

“We let (the patient) speak, we listen to children, what they think about it (their disease and treatment plan), more than before, it requires more time, … and parents who have some expectations, read something on the internet, so we together verify this.”.

*(HCP4)*

These quotes suggest that, after PCC training, professionals perceived that they were consciously working to include patients in the rehabilitation team, thus patients and professionals become partners.

Parents and children also perceived the change in approach towards one of inclusivity. One parent felt better prepared to negotiate the treatment plan via a PCC approach, as illustrated below:

“When it turned out that something was wrong with our daughter’s spine, we read on the Internet and talked to various specialists. After examination, the X-ray, everyone tried to convince us of the “right” therapy. We were confused. But no one really gave us enough time, or explained the mechanism of scoliosis, or the advantages and disadvantages of each therapy. Only in Ameryka (city in which Voivodeship Rehabilitation Hospital for Children is located). Previously, (in other facilities) nobody asked about our feelings or wanted to agree on an approach with us. Nowhere else we were able to discuss our concerns with the team of specialists. Knowing more about scoliosis, it was easier for us to talk to specialists and make decisions (about the treatment plan).”

*(P1)*

As part of the PCC intervention approach, the rehabilitation team offered information and the “Scoliosis Activity Diary”. The families were asked to read and bring their questions to the consultation. The elevation of the patient as an equal member of the team infers that the patient and his/her family must also take some responsibility for the treatment. This leads to the next core routine.

#### 3.2.2. Jointly Created Care Plans. Working the Partnership (PCC Routine 2)

After establishing a partnership via a PCC approach, the rehabilitation plan is co-created through a process of “negotiating” and “reaching a mutual agreement”. Enhanced patient engagement allows healthcare professionals to better understand patient limitations and capabilities, as well as functioning in their day-to-day environment. Information is shared, and together everyone discovers answers to the questions: What are their habits? How long can they wear the corset? What is their personality? Are they more persistent or the opposite? What is their level of activity? The recommended treatment plan involving many hours wearing the corset is often not well-received by patients, and the PCC approach was perceived to be helpful in making treatment more tolerable. One healthcare professional stated:

“We discuss with patients, we want to know what they think about it (proposed treatment) and how they assess it. If even there are tears at the beginning, that there is a corset on, then the discussion goes on, so that the patient begins to be interested in what’s next, what’s the outcome. That there are not only dark thoughts... that there is light in the tunnel, and that it will be better. (We discuss together with the child and the parents) what path to take for this child, so that (the treatment) meets their expectations and resolves the health problem.”

*(HCP8)*

Professionals reported that they perceived patients as more motivated to follow the plan, and expressed less worry about lack of adherence and poorer long-term outcomes. The patients and their families themselves described how much they appreciated the PCC approach. They reported increased satisfaction with care and improved adherence to the agreed upon treatment plan. One parent reported:

“I like this (PCC) approach, that medics are talking a lot with us, are really listening to and considering our suggestions. We really feel important … and that we have an influence.”

*(P2)*

A mother of another patient said:

“I like this control. I forgot to do something according to the treatment plan … actually, I procrastinated, but they called me, asked me how we are doing. Well, I was ashamed … but they motivated me. I did it quickly. Actually, I am happy about that.”

*(P3)*

#### 3.2.3. Safeguarding the Partnership (PCC Routine 3)

The third routine in the PCC approach requires documentation of the process of reaching a mutual agreement about the care plan and regularly reviewing and updating this documentation. In this PCC implementation pilot phase, professionals were, in most cases, willing to devote considerable time to listen to the patient narrative. Although documentation was available for a high percentage of PCC patients, documentation was not always comprehensive. Professionals reported that they struggled to identify key points from a patient’s narrative. Some examples of documentation from the open-ended PCC narrative field added to the EMR for this study included information such as: *“Child had a positive attitude to full-time corset treatment.” “Child has great support from family.” “Fear of wearing a corset to school.” “Fear of the reaction from others.” “A girl with an intellectual disability has a mother involved in treatment who will support the child.” “The patient does not wear a corset during school classes due to the nuisance of the corset.”*

These phrases represent a good start to the implementation of the PCC approach because they offer information that can be further explored in later sessions. Professionals expressed that they were willing to improve engagement in the PCC approach and its implementation in the hospital in the future. More training and individualized feedback could enhance implementation of this routine in later phases of implementation.

The process of mutual agreement about the treatment plan was perceived by rehabilitation professionals as increasing patient motivation to wear the corset. The PCC approach was also perceived to be helpful when challenges arose in carrying out the agreed upon care plan. The rehabilitation team learned to be more flexible and re-negotiate another care plan found to be more acceptable to the patient. A physiotherapist reported:

“Now (after implementation of PCC), one needs to pay more attention to talking with the patient about whether he/she accepts this method. Earlier ... yes, I might have asked, but in general, I approached the patient in such a way that I said ‘listen, we will practice with this method’ and that’s it. The patient did what I expected of him. However, now there is a conversation with the patient. If the patient does not accept it (the physiotherapeutic method), then I just change the method or ask a colleague (who is expert in other methods) for help, to show a patient how to work by another method (that is more acceptable to him/her).”

*(HCP9)*

### 3.3. Qualitative Data on Feasibility of Use of the Enablers in the WE-CARE Roadmap

The second purpose of the paper was to investigate the extent to which the WE-CARE Roadmap might support the implementation of PCC in a rehabilitation hospital in Poland. This pilot study provides initial evidence that the WE-CARE Roadmap may be useful to facilitate the implementation of PCC for patients with scoliosis in the Ameryka hospital, albeit not all enablers were addressed directly.

Adjustment of hospital infrastructure and the creation of new, PCC-specific fields in the hospital IT system resulted in increased interest in health care professionals in working according to PCC routines. Although the IT work to create and deploy the fields incurred some one-time costs, maintenance costs did not increase. Almost all PCC patients in this pilot study were asked to provide a narrative at many points during their care to different types of health care professional on the multi-disciplinary care team. Although the percentage of PCC fields completed is considered an accurate representation of PCC adherence, because the field aligned with each of the three PCC routines, the quality of the documentation in text fields may vary. Content will be closely examined in later phases of implementation in order to provide appropriate feedback and suggestions for improvement to health care professionals. These IT data fields offered a measurement system to monitor PCC fidelity and will assist professionals in improving their implementation of the PCC approach in the future.

Similarly, the “Scoliosis Activity Diary” was designed and implemented as an incentive, to enhance commitment to carry out the mutually agreeable care plan. All patients used the “Scoliosis Activity Diary”, which was perceived by professionals to facilitate their self-efficacy. Research suggests that the measurement process itself may lead to improvement of monitored indicators, e.g., [28], thus diary use may increase patients’ motivation.

Regarding incentives resulting from implementation of a PCC approach, one physician reported:

“What motivates me (to implement PCC) are the outcomes of treatment. … that a patient adheres more to the treatment plan. That they participate, this (co-creation of treatment plan) is not next to them. Well, (earlier) I had a feeling, that I am saying something (about the treatment), the parent goes out of their way (be driven round the bend), but the patient (behaves) as if he was outside (that the situation does not relate to them).”

*(HCP5)*

And one mother reported:

“I see how my daughter is now engaged in the following of the therapy. She very scrupulously wears the corset, and logs her hours (in the ‘Activity Diary’). She is doing this for herself, but she can also show this to me and the doctor.”

*(P2)*

Future phases of implementation will focus more on contracting strategies as an enabler, and a more accurate capture of cost containment. Previous studies suggest that PCC improves the quality of treatment and improves cost containment [3,22,29]. For this patient population, rehabilitation is reimbursed by a public payer as a lump sum for services received. Quality of treatment and medical outcomes are not included in the reimbursement criteria. The current contracting strategies incentivize the number of services delivered and may therefore result in an increase in spending. At the hospital level, the introduction of PCC did not directly reduce costs in the pilot phase, but there is some indication of cost containment at the healthcare system level. Professionals reported that they were less likely to hear of patients reporting seeking a second opinion in other medical centers and, comparing this to before the PCC was introduced in the hospital, they concluded that it resulted from the trust that the PCC approach generated. Future savings might also result from systematic implementation of PCC, as one physician stated:

“We prescribe a corset, and it is made. The patient goes somewhere, someone says that this method is not so necessary. The patient is not motivated enough, so they listen to what suits them and reject the corset. (Public) money is spent on this corset, for this treatment, there are, so, … financial losses for this orthopedic equipment. Actually double losses, because he went somewhere else to another clinic. And as a consequence, … well, they come to us, let’s say after a year, ... We have such a patient now. Her health has deteriorated, she has had a significant progression of scoliosis. The corset was made and thrown out. A new one needs to be made, and possibly even a scoliosis surgery would need to be performed because the scoliosis is already so advanced that it requires much more money than the corset itself.”

*(R3)*

Based on these perspectives, there are two mechanisms through which systemic implementation of PCC might reduce or contain healthcare system costs: (1) by increasing concordance in treatment between patient and physician and preventing disease progression that results in higher future costs to the healthcare system and (2) saving unnecessary healthcare expenses related to obtaining a second opinion. The net result may be cost containment in the health care system, but such documentation would require a measurement system capable of tracking quality and costs across the whole chain of care. Moreover, better adherence has been shown to lead to improved medical outcomes, and this may reduce costs for future medical services such as avoided surgical operations on the spine, treatment of later pain in adulthood as well as improved quality of life [19]. Verification of such findings will require long-term follow-up of this patient population, which is planned for future study.

## 4. Conclusions

In summary, this pilot feasibility study indicates that the PCC approach used in Sweden can be successfully transferred to a rehabilitation hospital in Poland and that the application of the WE-CARE Roadmap helped to facilitate the implementation process [1,23]. As a result, professionals, patients and their families expressed favorable perceptions of implementation. They regarded the PCC approach as feasible and endorsed it as beneficial. Future phases of implementation will improve monitoring and feedback and incorporate new enablers into the implementation strategy with improved measurement systems to capture care quality and costs throughout the care continuum.

## Figures and Tables

**Table 1:** Quantitative person-centered care (PCC) measures related to PCC implementation effectiveness in the pilot phase. EMR = electronic medical record.

Number of PCC Patients	Percent of Patients with a Narrative Summary in the EMR by Type of Health Care Professional				Percent with a Documented Treatment Plan	Percent of Patients Using the Activity Diary
51	Physician	Physiotherapist	Nurse	Psychologist	86% (*n* = 44)	100% (*n* = 51)
	92% (*n* = 47)	100% (*n* = 51)	98% (*n* = 50)	98% (*n* = 50)		

## Data Availability

The data presented in this study are available on request from the corresponding author. The data are not publicly available due to privacy reasons.

## Supplementary Materials

The following are available online at https://www.mdpi.com/1660-4601/18/5/2205/s1, Booklet S1: Polish-language version of the “Scoliosis Activity Diary”.
