# Supplementary material for: Implementation of Person-Centered Care: A Feasibility Study Using the WE-CARE Roadmap
Source: Int J Environ Res Public Health. 2021 Feb 24;18(5):2205. doi: 10.3390/ijerph18052205 (PMC7956736; doi:10.3390/ijerph18052205)
Supplement: Supplementary file 1 [file ijerph-18-02205-s001.pdf]

AMERYKA

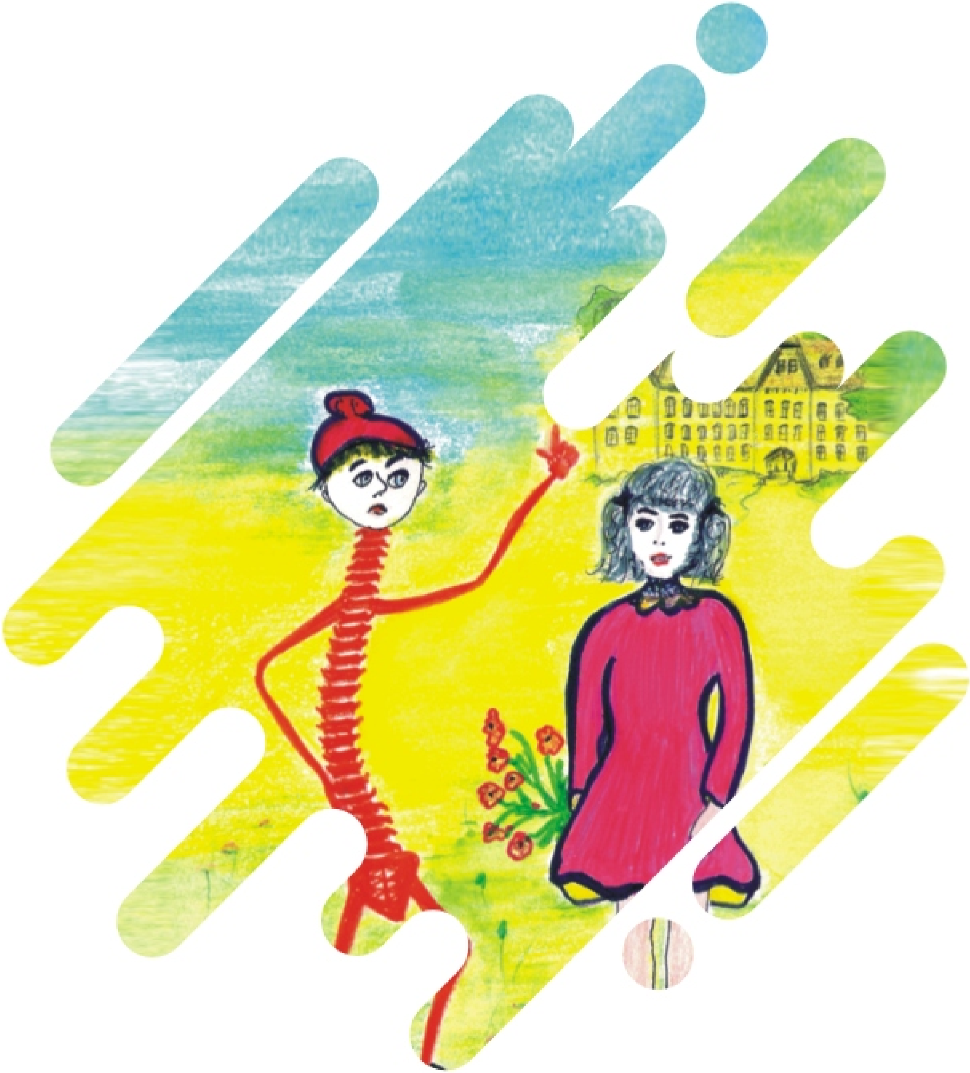

Imię i Nazwisko

Adres

Telefon

Wydane przez:
Wojewódzki Szpital Rehabilitacyjny dla Dzieci w Ameryce k/Olsztynka
Ilustracje: Urszula Serafińska
Autor tekstu: Agnieszka Adamiak - Morska

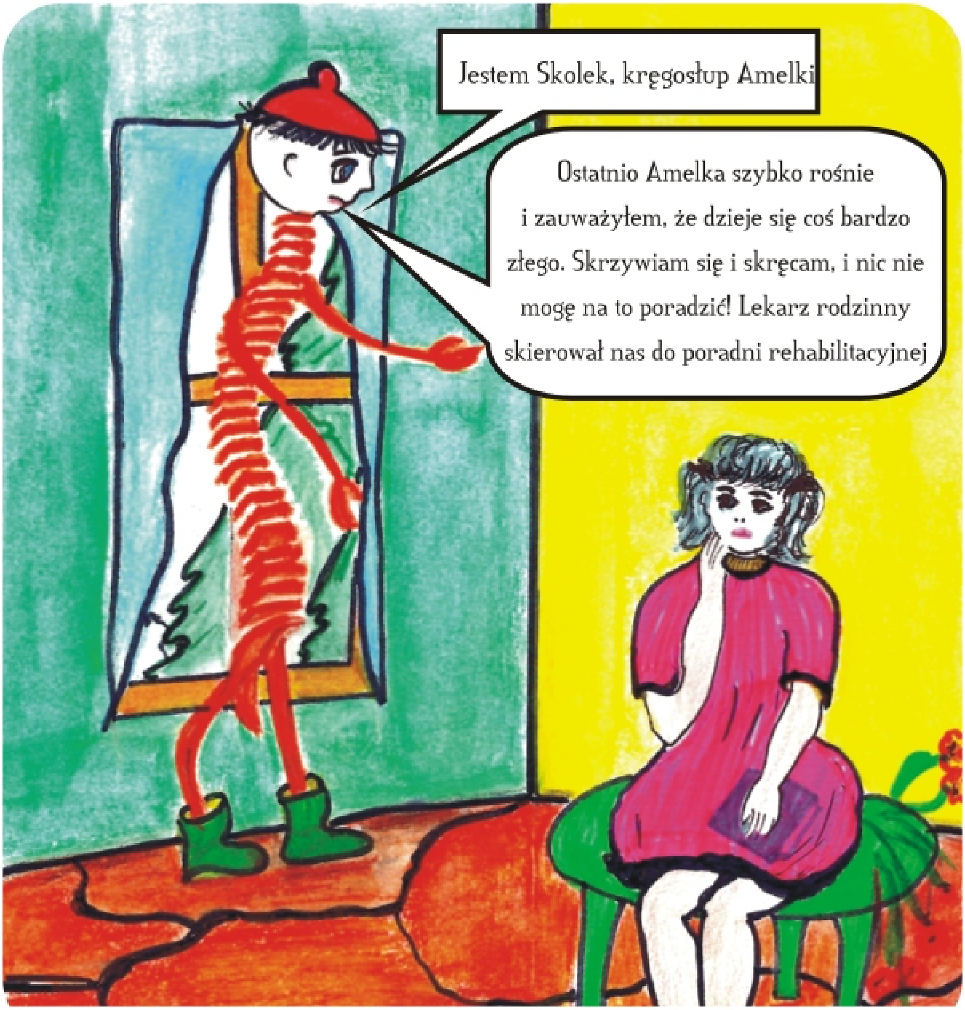

Skolioza nie jest jedynie bocznym skrzywieniem kręgosłupa, ale jest deformacją trójpłaszczyznową, na którą składają się:

- w płaszczyźnie czołowej: skrzywienie boczne przekraczające 10°
- w płaszczyźnie strzałkowej: spłycenie lub pogłębienie naturalnie występujących krzywizn kręgosłupa, tzw. kifo - lub lordoskoliozy
- w płaszczyźnie poprzecznej: rotacja i torsja kręgów.

Konsekwencją złożonego charakteru wady jest znaczna deformacja sylwetki oraz trudności w leczeniu i konieczność doboru indywidualnej terapii w zależności od charakteru i stopnia zaawansowania choroby.

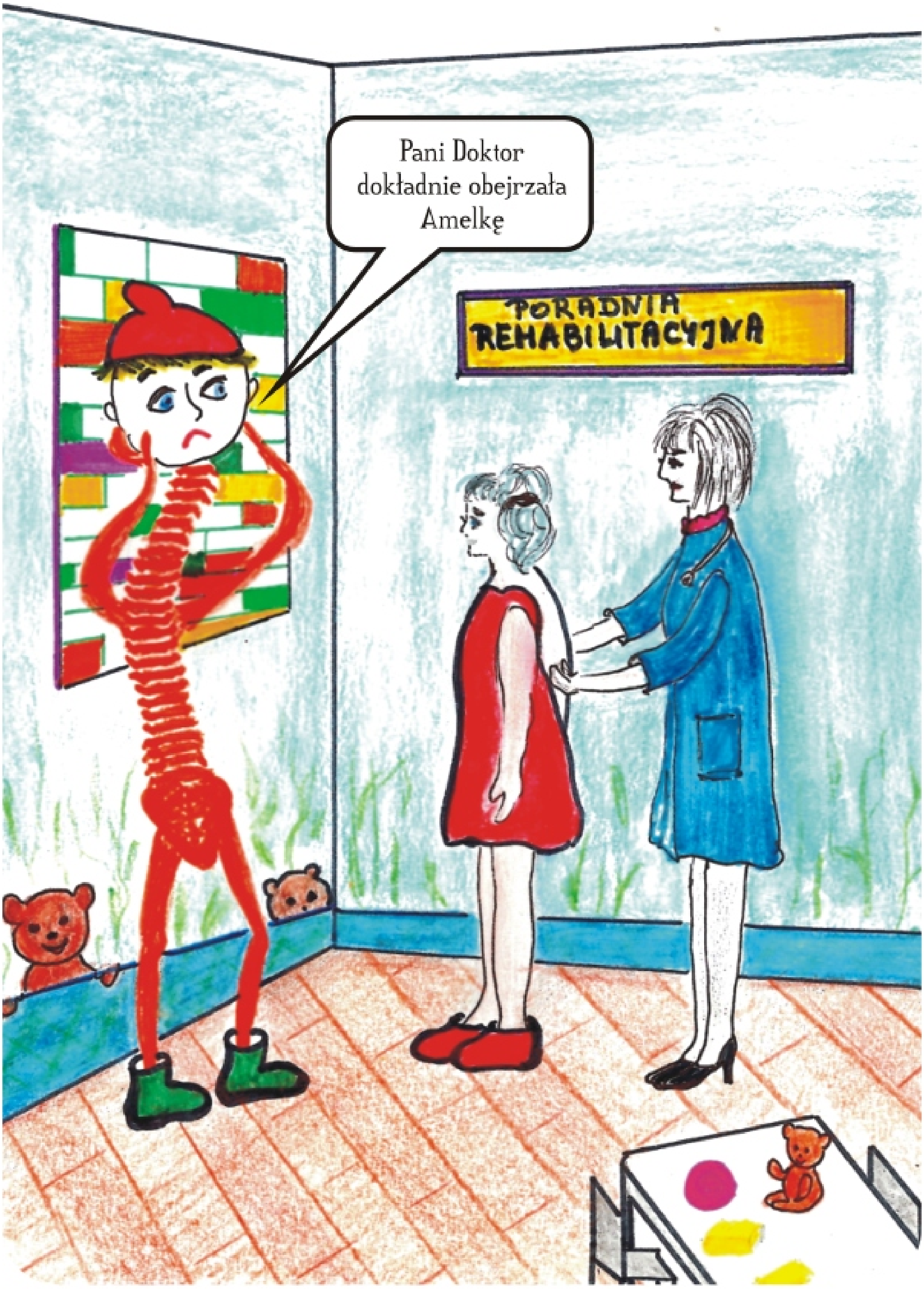
Pani Doktor dokładnie obejrzała Amelkę
PORADNIA REHABILITACYJNA

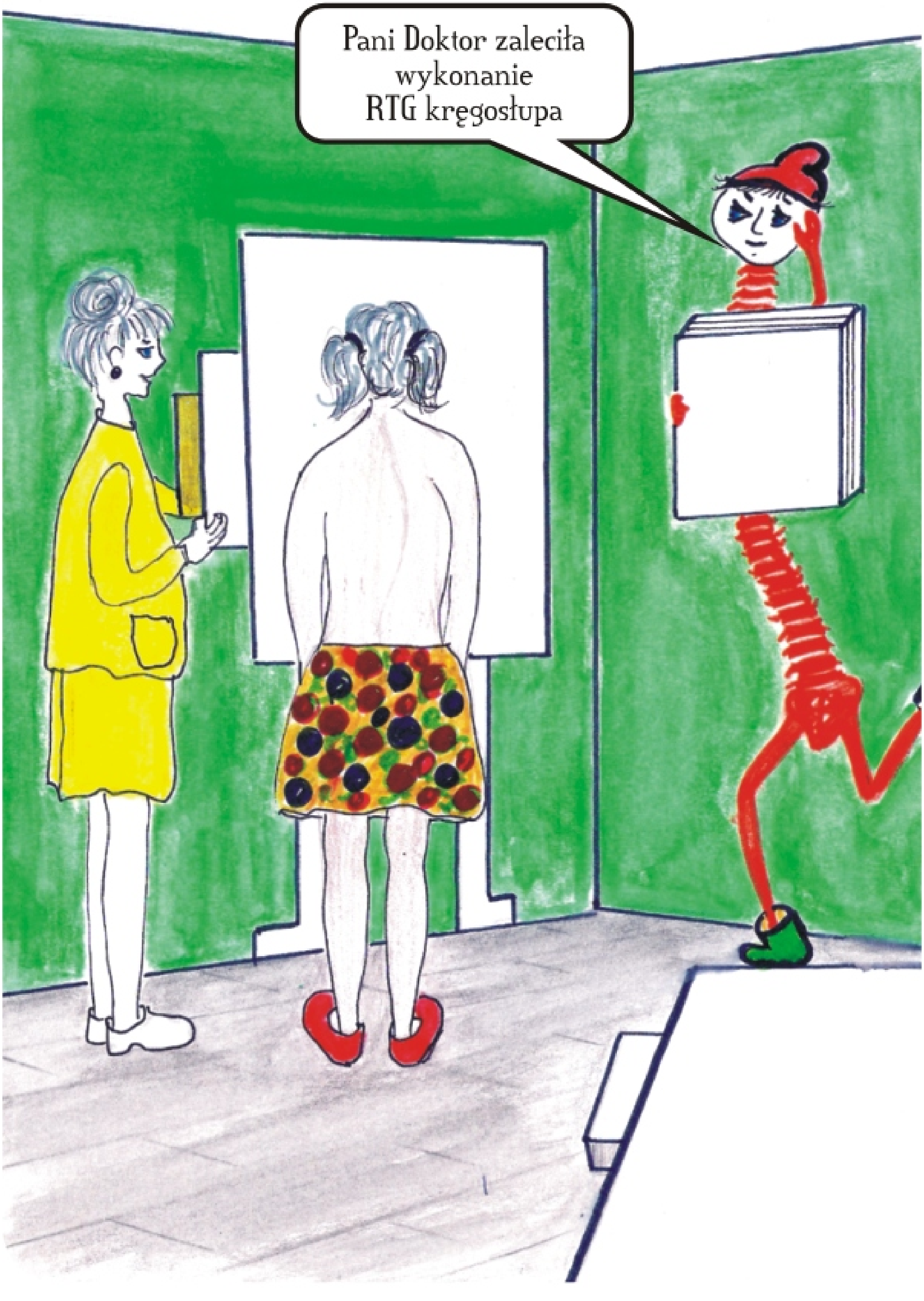
Pani Doktor zaleciła
wykonanie
RTG kręgosłupa

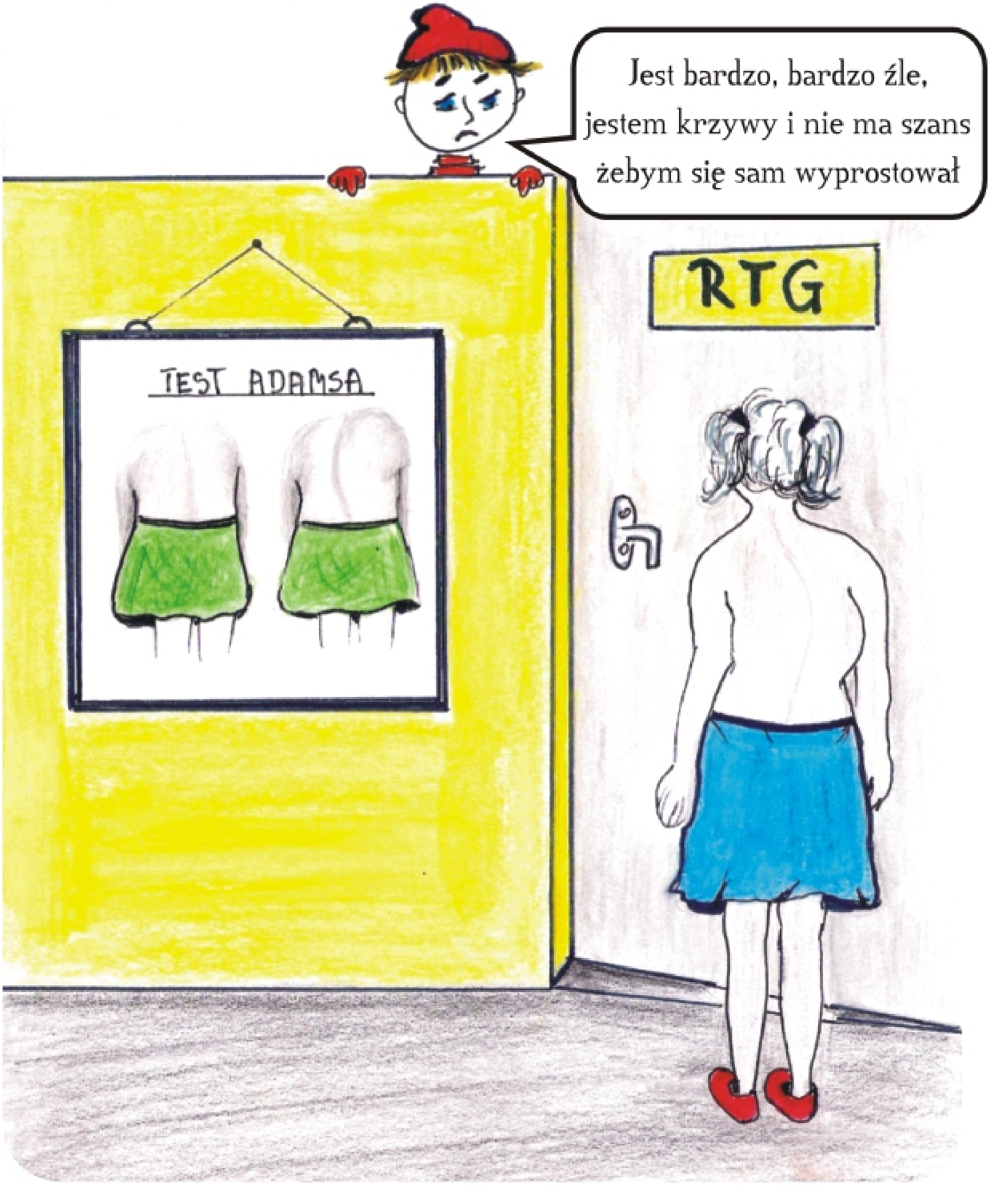

Zgodnie z rekomendacją SOSORT czyli Międzynarodowego Towarzystwa Ortopedycznego i Rehabilitacyjnego Leczenia Skolioz, przy skoliozach przekraczających 20° zalecanym leczeniem jest terapia łącząca specyficzne ćwiczenia ze stosowaniem gorsetu ortopedycznego.

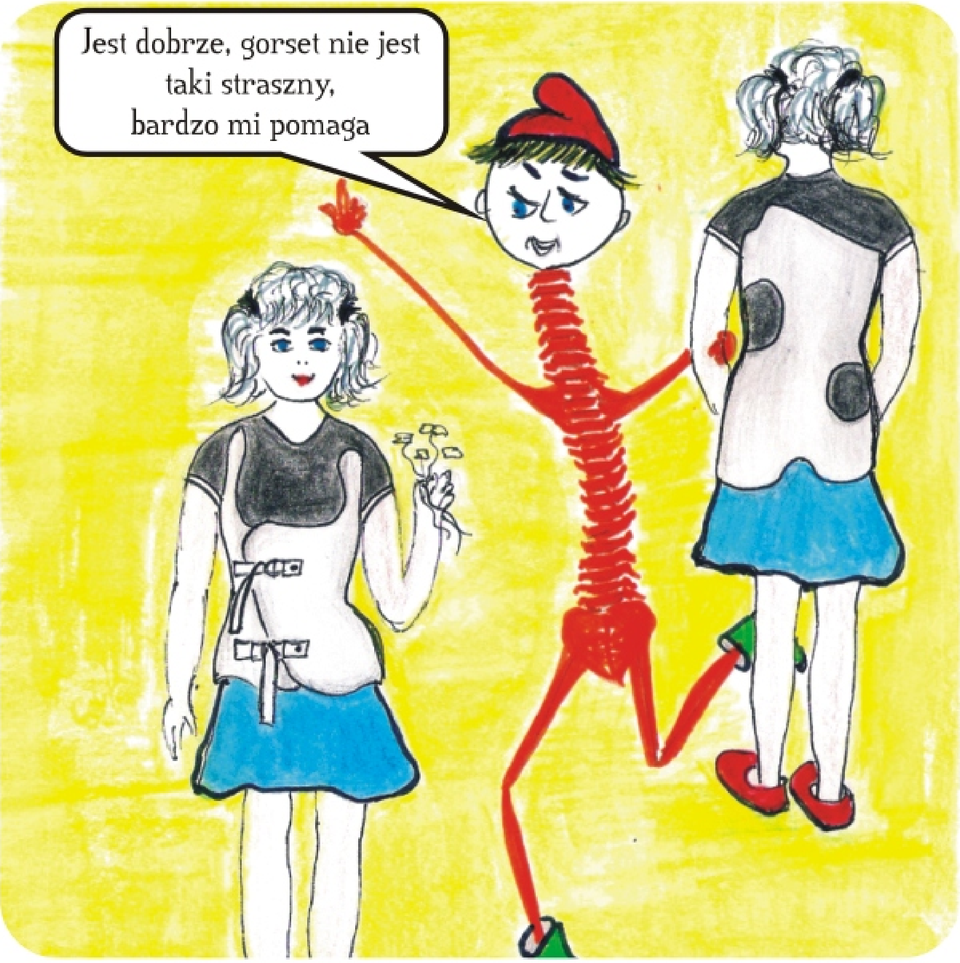

Działanie gorsetu ortopedycznego jest złożone:

- z jednej strony poprzez odpowiednio dobrane strefy nacisku powoduje przesunięcie tkanek od wypukłości do wklęsłości
- wydłuża kręgosłup, powoduje derotację klatki piersiowej i kręgów, jest to tzw. działanie bierne gorsetu
- w czasie leczenia gorsetem obserwujemy aktywację czynnych mechanizmów korygujących deformację tułowia. Zaliczamy do nich:
  - korekcyjny wpływ w czasie wzrastania,
  - asymetryczne ruchy oddechowe korygujące klatkę piersiową,
  - przywrócenie prawidłowego przebiegu mięśni grzbietu,
  - aktywizacja układu posturalnego w prawidłowej orientacji przestrzennej.

ZALECENIA

1. Kontynuuj wyuczone ćwiczenia przez 30 minut dziennie.
2. Zgłoś się do zakładów ortopedycznych w celu wykonania gorsetu ortopedycznego.
3. Zakładaj gorset w pozycji leżącej, na bawełnianą bieliznę.
4. Noś gorset docelowo 20 h/ dobę.
5. Wykonuj ćwiczenia oddechowe w gorsecie.
6. Koryguj postawę podczas wykonywania czynności dnia codziennego bez gorsetu.
7. Zgłoś się do poradni rehabilitacyjnej miesiąc po wykonaniu gorsetu celem kontroli gorsetu, uzyskania instruktażu użytkowania gorsetu i ewentualnie skierowania na zdjęcie Rtg kręgosłupa w gorsecie.
8. Systematycznie zgłaszaj się do kontroli w poradni rehabilitacyjnej co 3 m-ce lub w razie potrzeby.
9. Ćwicz regularnie w domu, a okresowo pod opieką fizjoterapeuty w Szpitalu Rehabilitacyjnym w Ameryce.
10. Nie rezygnuj z lekcji wychowania fizycznego.
11. Uprawiaj sporty w porozumieniu z lekarzem i fizjoterapeutą.

KORECJA

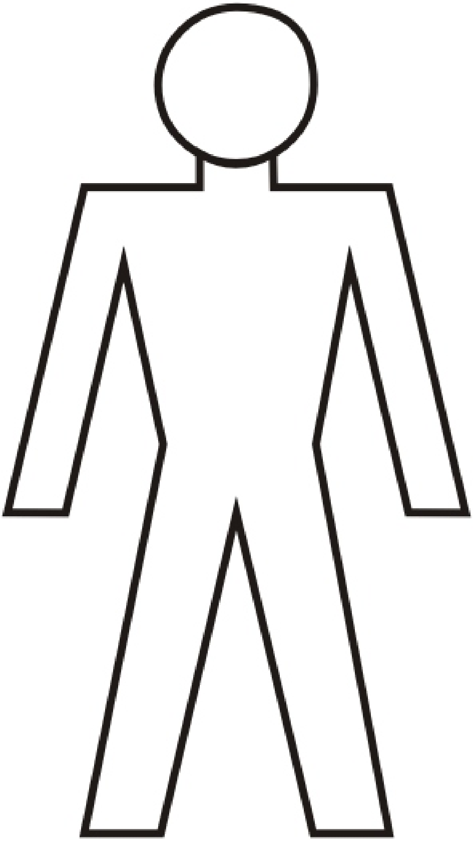

KOREKCJA

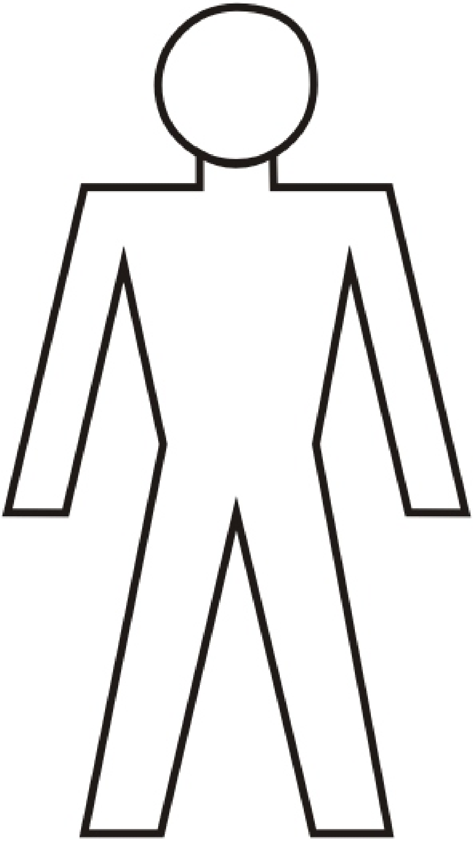

KOREKCJA

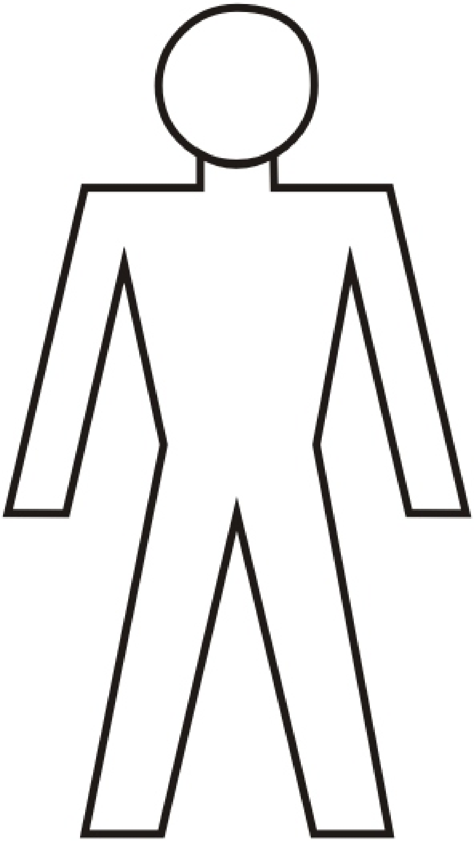

DZIENNICZEK AKTYWNOŚCI

MIESIĄC……………………………………………………………………..

DZIEŃ 1	DZIEŃ 2	DZIEŃ 3	DZIEŃ 4	DZIEŃ 5	DZIEŃ 6	DZIEŃ 7

DZIEŃ 8	DZIEŃ 9	DZIEŃ 10	DZIEŃ 11	DZIEŃ 12	DZIEŃ 13	DZIEŃ 14

DZIEŃ 15	DZIEŃ 16	DZIEŃ 17	DZIEŃ 18	DZIEŃ 19	DZIEŃ 20	DZIEŃ 21

DZIEŃ 22	DZIEŃ 23	DZIEŃ 24	DZIEŃ 25	DZIEŃ 26	DZIEŃ 27	DZIEŃ 28

DZIEŃ 29	DZIEŃ 30	DZIEŃ 31

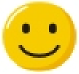 Ćwiczę i noszę gorset

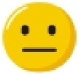 Tylko ćwiczę/ tylko noszę gorset

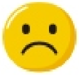 Nic nie zrobiłam/em

*Zaznacz odpowiednią odpowiedź

DZIENNICZEK AKTYWNOŚCI

MIESIĄC……………………………………………………………………

DZIEŃ 1	DZIEŃ 2	DZIEŃ 3	DZIEŃ 4	DZIEŃ 5	DZIEŃ 6	DZIEŃ 7
**DZIEŃ 8**	**DZIEŃ 9**	**DZIEŃ 10**	**DZIEŃ 11**	**DZIEŃ 12**	**DZIEŃ 13**	**DZIEŃ 14**
**DZIEŃ 15**	**DZIEŃ 16**	**DZIEŃ 17**	**DZIEŃ 18**	**DZIEŃ 19**	**DZIEŃ 20**	**DZIEŃ 21**
**DZIEŃ 22**	**DZIEŃ 23**	**DZIEŃ 24**	**DZIEŃ 25**	**DZIEŃ 26**	**DZIEŃ 27**	**DZIEŃ 28**
**DZIEŃ 29**	**DZIEŃ 30**	**DZIEŃ 31**				

Ćwiczę i noszę gorset

Tylko ćwiczę/ tylko noszę gorset

Nic nie zrobiłam/em

*Zaznacz odpowiednią odpowiedź

DZIENNICZEK AKTYWNOŚCI

MIESIĄC……………………………………………………………………….

DZIEŃ 1	DZIEŃ 2	DZIEŃ 3	DZIEŃ 4	DZIEŃ 5	DZIEŃ 6	DZIEŃ 7

DZIEŃ 8	DZIEŃ 9	DZIEŃ 10	DZIEŃ 11	DZIEŃ 12	DZIEŃ 13	DZIEŃ 14

DZIEŃ 15	DZIEŃ 16	DZIEŃ 17	DZIEŃ 18	DZIEŃ 19	DZIEŃ 20	DZIEŃ 21

DZIEŃ 22	DZIEŃ 23	DZIEŃ 24	DZIEŃ 25	DZIEŃ 26	DZIEŃ 27	DZIEŃ 28

DZIEŃ 29	DZIEŃ 30	DZIEŃ 31

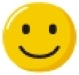
Ćwiczę i noszę gorset

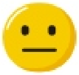
Tylko ćwiczę/ tylko noszę gorset

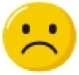
Nic nie zrobiłam/em

*Zaznacz odpowiednią odpowiedź

DZIENNICZEK AKTYWNOŚCI

MIESIĄC………………………………………………………………………

DZIEŃ 1	DZIEŃ 2	DZIEŃ 3	DZIEŃ 4	DZIEŃ 5	DZIEŃ 6	DZIEŃ 7
☐	☐	☐	☐	☐	☐	☐
DZIEŃ 8	DZIEŃ 9	DZIEŃ 10	DZIEŃ 11	DZIEŃ 12	DZIEŃ 13	DZIEŃ 14
☐	☐	☐	☐	☐	☐	☐
DZIEŃ 15	DZIEŃ 16	DZIEŃ 17	DZIEŃ 18	DZIEŃ 19	DZIEŃ 20	DZIEŃ 21
☐	☐	☐	☐	☐	☐	☐
DZIEŃ 22	DZIEŃ 23	DZIEŃ 24	DZIEŃ 25	DZIEŃ 26	DZIEŃ 27	DZIEŃ 28
☐	☐	☐	☐	☐	☐	☐
DZIEŃ 29	DZIEŃ 30	DZIEŃ 31				
☐	☐	☐				

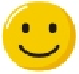 Ćwiczę i noszę gorset

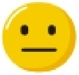 Tylko ćwiczę/ tylko noszę gorset

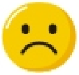 Nic nie zrobiłam/em

*Zaznacz odpowiednią odpowiedź

DZIENNICZEK AKTYWNOŚCI

MIESIĄC..

DZIEŃ 1	DZIEŃ 2	DZIEŃ 3	DZIEŃ 4	DZIEŃ 5	DZIEŃ 6	DZIEŃ 7

DZIEŃ 8	DZIEŃ 9	DZIEŃ 10	DZIEŃ 11	DZIEŃ 12	DZIEŃ 13	DZIEŃ 14

DZIEŃ 15	DZIEŃ 16	DZIEŃ 17	DZIEŃ 18	DZIEŃ 19	DZIEŃ 20	DZIEŃ 21

DZIEŃ 22	DZIEŃ 23	DZIEŃ 24	DZIEŃ 25	DZIEŃ 26	DZIEŃ 27	DZIEŃ 28

DZIEŃ 29	DZIEŃ 30	DZIEŃ 31

Ćwiczę i noszę gorset

Tylko ćwiczę/ tylko noszę gorset

Nic nie zrobiłam/em

*Zaznacz odpowiednią odpowiedź

DZIENNICZEK AKTYWNOŚCI

MIESIĄC……………………………………………………………………

DZIEŃ 1	DZIEŃ 2	DZIEŃ 3	DZIEŃ 4	DZIEŃ 5	DZIEŃ 6	DZIEŃ 7
DZIEŃ 8	DZIEŃ 9	DZIEŃ 10	DZIEŃ 11	DZIEŃ 12	DZIEŃ 13	DZIEŃ 14
DZIEŃ 15	DZIEŃ 16	DZIEŃ 17	DZIEŃ 18	DZIEŃ 19	DZIEŃ 20	DZIEŃ 21
DZIEŃ 22	DZIEŃ 23	DZIEŃ 24	DZIEŃ 25	DZIEŃ 26	DZIEŃ 27	DZIEŃ 28
DZIEŃ 29	DZIEŃ 30	DZIEŃ 31				

Ćwiczę i noszę gorset

Tylko ćwiczę/ tylko noszę gorset

Nic nie zrobiłam/em

*Zaznacz odpowiednią odpowiedź

PAMIĘTAJ!

Pracując wytrwale osiągniesz zamierzony cel.

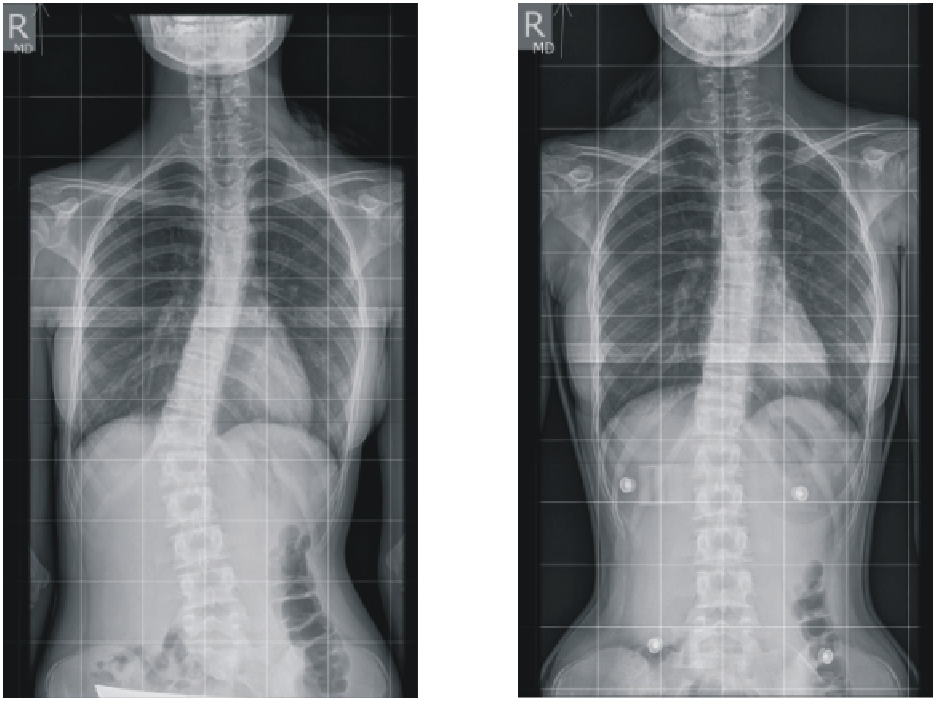

zdj. z archiwum szpitalnego

Ameryka 21, 11-015 Olsztynek

89 519 48 23

szpital@ameryka.com.pl

www.ameryka.com.pl
